# An extremely rare case of lesser omental hernia in an elderly female patient following total colectomy

**DOI:** 10.1186/s12893-019-0665-7

**Published:** 2020-01-16

**Authors:** Zhicheng Liu, Liang He, Yan Jiao, Zhonghang Xu, Jian Suo

**Affiliations:** 1 Department of Gastrointestinal Surgery, The First Hospital of Jilin University, 71 Xinmin Ave., Changchun, 130021 Jilin China; 2 Department of Hepatobiliary and pancreatic Surgery, The First Hospital of Jilin University, Changchun, 130021 Jilin China; 3 Department of Gastrointestinal Surgery, China-Japan Union Hospital of Jilin University, Changchun, 130021 Jilin China

**Keywords:** Internal hernia, Lesser omental hernia, Intestinal obstruction, Total colectomy

## Abstract

**Background:**

An intro-abdominal hernia through the lesser omentum is a rare but severe condition that can cause intestinal obstruction and other life-threating complications. Until now, only a handful of cases have been reported worldwide. The diagnosis of lesser omental hernia remains challenging for emergency surgeons because of the unspecific symptoms. Therefore, there is a need for a better understanding of the characteristics of this condition.

**Case presentation:**

In this report, we described the case of a 73-year-old female patient who was diagnosed with a lesser omental hernia caused by previous total colectomy. The patient underwent emergency surgery, and the intraoperative findings revealed a 200-cm segment of the small intestine was herniated through a defected lesser omentum (approximately 3 × 4 cm) from the lesser retrogastric curvature of the stomach. Besides, we summarize the specific abdominal computed tomography (CT) findings of lesser omental hernia by reviewing the literature.

**Conclusion:**

The lesser omental hernia is extremely rare but can cause serious complications. The cause of lesser omental hernia can be congenital or acquired. Careful examination of the small omentum before the closure of the abdomen is expected to reduce the occurrence of these abdominal surgery-associated complications. The specific features of abdominal CT in cases of lesser omental hernia, which are summarized in this article, can help other clinicians to obtain accurate diagnoses of lesser omentum hernia in the future.

## Background

The lesser omental hernia is a rare form of intestinal hernia. However, it is a severe clinical condition and can result in intestinal obstruction and other life-threatening complications [1, 2]. To date, very few cases of lesser omental hernia have been reported across the world [3–13]. It is challenging for emergency surgeons to make a timely and accurate diagnosis of lesser omental hernia, as the symptoms are unspecific and overlap with those of other gastrointestinal diseases. Therefore, a better understanding of the characteristics of lesser omental hernia is needed.

In this study, we describe an uncommon case of lesser omental hernia in a 73-year-old female patient that appeared to be mainly caused by a total colectomy that was performed 2 years earlier. We also conduct a literature review on the lesser omental hernia.

## Case presentation

### Patient description

An elderly female patient, aged 73-year-old, was admitted to our hospital for unexplained acute abdominal pain and bloating after the occurrence of vomiting. Upon admission, the patient was observed to have an abdominal bulge and total abdominal tenderness accompanied by rebound pain and muscle tension. The patient had a medical history of total colectomy in combination with an ileal pouch-anal anastomosis (IPAA) for the treatment of functional chronic constipation or chronic idiopathic constipation (CIC) 2 years before hospital admission.

### Examinations and diagnosis

**Fig. 1:**
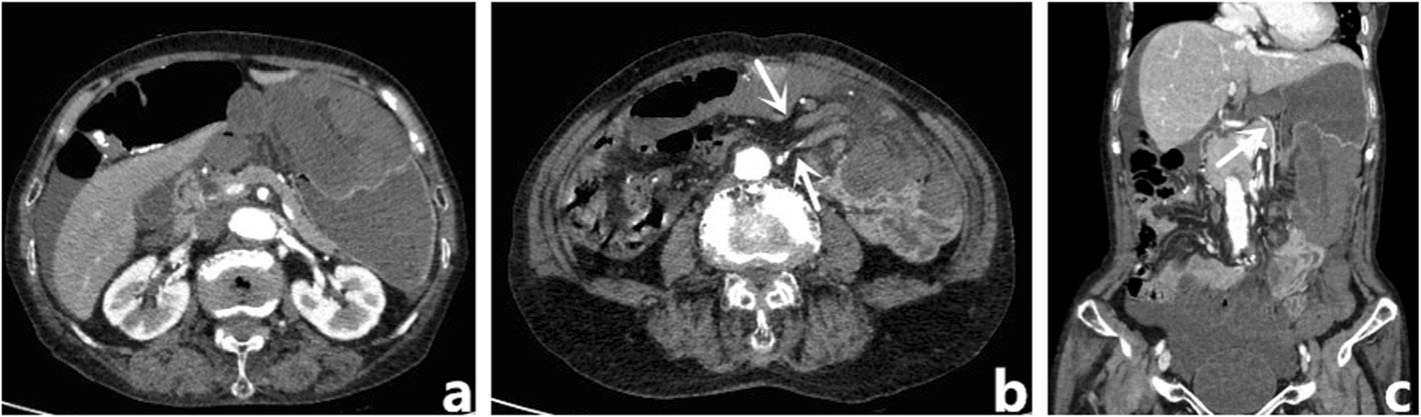
Abdominal CT images. **a** There is distention of the small intestine in the distal, ventral part of the distorted stomach, accumulated liquid around the liver; and reduced enhancement in the intestinal mucosa; (**b**) The small intestine is prolapsed through the defected lesser omentum, as denoted by the arrows; (**c**) These is dislocation of the blood vessels of the small intestine and distorted stomach, as indicated by arrow

General physical examinations were as follows: heart rate: 120 bpm; blood pressure: 85/50 mmHg; Laboratory tests revealed a white blood cell (WBC) count of 17 × 10^9^ / L, and lactic acid (LA) level of 6.3 mmol / L; Abdominal enhanced computed tomography (CT) showed dilation, as well as gas and liquid accumulation in the distal portion of the small intestine, ventral to the distorted stomach. It was observed that a segment of the small intestine was prolapsed via the defected lesser omentum, passing the retrogastric lesser curvature to enter the ventral part of the stomach. CT images in the abdomen also revealed a hypodensity in some fragments of the small intestine, liquid accumulation around the liver and spleen, as well as pelvic effusion (Fig. 1).

Based upon the abdominal CT findings, the patient was diagnosed with having a lesser omental hernia complicated with intestinal obstruction, acute diffuse peritonitis, and septic shock.

### Treatment

**Fig. 2:**
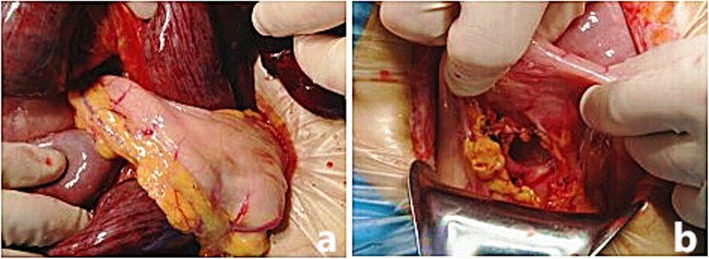
Images of the findings during laparotomy. **a** A 200-cm segment of the small intestine protruded from the lesser retrogastric curvature of the stomach to a defected lesser omentum; (**b**) A defected lesser omentum, of approximately 3 × 4 cm in size, was visualized

The patient was immediately treated with an emergency laparotomy. During the procedure, ascites, of approximately 1500 ml liquid with blood, were observed. Notably, a fragment of the small intestine protruded from the lesser retrogastric curvature of the stomach into a defected lesser omentum (Fig. 2a) and appeared necrotic and black. The defected omentum was approximately 3 × 4 cm in size (Fig. 2b), with the necrotic fragment of the small intestine measuring approximately 200-cm in length. The necrotic portion of the small intestine was resected, anastomosis was performed, and the defected lesser omentum was closed. The patient was subsequently transferred to the intensive care unit (ICU). The tracheal intubation was removed 2 days after the operation, and the patient was transferred to a local hospital for further treatment.

The risks and benefits of the surgical procedures were explained to the patient, and written informed consent was obtained.

## Discussion and conclusion

Intra-abdominal hernias represent a rare type of internal hernia, accounting for approximately 1% of all internal hernias [1]. This type of hernia is characterized by the protrusion of organs in the abdomen through normal or abnormal openings (pouches) into the abdominal cavity. Intra-abdominal hernias are categorized according to the cause as either congenital intra-abdominal hernia (e.g., duodenal fistula, Winslow hernia) or acquired intra-abdominal hernia, caused by surgery, trauma, inflammation, and other factors that can result in defects in the greater omentum or lesser omentum. Compared with greater omentum hernia, lesser omentum hernia is considerably rarer, with only a few reported cases. Kitagishiet and colleagues classified lesser omental hernias into two types: type I and type II. In type I lesser omentum hernia, the intestine prolapses directly into the lesser omental sac through the lesser omentum, whereas, in type II, lesser omentum hernia, the gastrocolic ligament and the small intestine prolapses from the retrogastric curvature into the abdominal cavity through the lesser omentum. The present case was classified as type II lesser omental hernia, based on the main intraoperative finding that the small intestine prolapsed from the lesser retrogastric space of the stomach through a defected lesser omentum into the abdominal cavity. Furthermore, the patient had a medical history of total colectomy but had no evidence of inflammation or adhesion around the defected lesser omentum. She did not have a history of trauma or congenital abnormality. The defect in the lesser omentum in our patient was likely caused by the previous total colectomy.

Additionally, we performed a literature search using the keyword “lesser omental hernia” and obtained a total of 11 reports in PubMed. The findings of these cases, including ours, are summarized in Table 1 [3–13]. The mean age of the patients was 43.5 years, ranging from 14 to 73 years. The 11 previously reported cases and our case of lesser omental hernia consisted of six males and six females. Type I patterns were observed in five patients, while type II patterns were found in seven patients. The patient in the present study was defined as having type II lesser omental hernia. Analysis of causative factors indicated that defects in the lesser omentum were caused by congenital factors in six cases [3–6, 9, 10], while 3 cases were caused by previous abdominal surgeries [7, 8], and the remaining cases were caused by unidentified factors. Our patient underwent a total colectomy approximately 2 years before the onset of the lesser omental hernia. Notably, similarities were observed in the abnormal anatomical basis for the development of lesser omental hernia between Case 7 and Case 11, Case 4 and Case 8, Case 5, and the present case. Because of this, Type II lesser omental hernia was further divided into the following three subtypes: (1) Type II a is characterized by a combined defect in the ligamenta gastrocolicum. Case 7 and Case 11 are classified as being Type II a; (2) Type II b is characterized by a combined defect in the mesocolon transversum. Case 4 and Case 8 were defined as being Type II b; (3) Type II c is characterized by openness in the posterior wall of the omental sac. Case 5 and the present were classified as being Type II c. It merits the attention that the defects in Type II c lesser omental hernia is likely to be caused by previous abdominal surgery, resulting in an abnormal anatomic basis for the formation of the lesser omental hernia. Therefore, it is highly recommended that surgeons carefully examine the small omentum before the closure of the abdomen in abdominal surgery, to avoid postoperative complications, such as the lesser omentum hernia, as observed in the present case.

**Table 1:** Summary of previously reported cases with lesser omental hernia

Case	Refs.	Year	Age	Gender	Type	History of abdominal surgery	Intraoperative findings	CT findings	Herniated organ	Surgical method	
										Bowel resection	Orifice closure
1	Li A**[**3**]**	2017	38	F	I	–	Malrotation of the midgut, dissociation of Ileocecal, endometriosis	No CT examination	Ileum	+	+
2	Rathnakar SK**[**4**]**	2016	54	M	I	–	Defect in the lesser omentus (3 × 2 cm in size)	No CT examination	Jejunum	–	+
3	Wang W**[**5**]**	2016	62	F	I	–	Defects in ligamentum hepatogastricum (3 × 2 cm in size) complicated with defects in the mesocolon transversum, greater omentum,	Small intestine and mesostenium vessels gathered in the lesser gastric curvature	Jejunum	+	+
4	Kundaragi NG**[**6**]**	2014	55	M	II	–	Defects in ligamentum hepatogastricum (4 cm in diameter), complicated with defect in the mesocolon transversum	Distorted stomach became thinner and longer; colon transversum was relocated, dilated bowel loops were visible in the ventral part of the stomach	Small intestine	–	+
5	Konishi T**[**7**]**	2014	42	M	II	Total colectomy	Defect in the lesser omentum (5 cm in diameter)	air-fluid levels above the stomach dilated bowel loops were located in the ventral part of the stomach and the mesostenium vessels gathered in the lesser curvature of the stomach	Jejunum	–	+
6	Masubuchi S**[**8**]**	2013	57	F	II	Left hemi-colectomy	Defects in the ligamentum hepatogastricum (4 cm in diameter), complicated with the mesocolon transversum (5 cm in diameter)	Distorted stomach, obstructed bowel loops located in the ventral part of the distal stomach	Ileum	–	+
7	Min JS**[**9**]**	2009	47	F	II	–	Defects in ligamentum hepatogastricum and ligamenta gastrocolicum	Dilated jejunum with liquid accumulation, mesostenium perforated through the defected greater omentum	Jejunum	+	+
8	Bahadori K**[**10**]**	2001	14	F	II	Appendectomy	Defects in the lesser omentum and mesocolon transversum	Distortion and relocation of the stomach	Small intestine		
9	Duarte GG**[**11**]**	1996	36	M	I	Unknown	Defect in the lesser omentum	No CT examination	Ileum	+	+
10	Tran TL**[**12**]**	1990	24	M	I	Unknown	Defect in ligamentum hepatogastricum (4 cm in diameter)	Dilated bowel loops were visible within the omental burs, the spleen was relocated.	Jejunum	+	+
11	Yasuda S**[**13**]**	1984	20	M	II	Unknown	Defects in ligamentum hepatogastricum (4 cm in diameter) and ligamenta gastrocolicum	Upper abdominal air-fluid levels	Small intestine	+	+
12	Current case	2018	73	F	II	Subtotal colectomy	Defect in ligamentum hepatogastricum (4 cm in diameter)	Distorted stomach, perforated mesostenium through the defected lesser omentum, dilated bowel loops located in the ventral part of the distal stomach and relocated mesostenium vessels	Jejunum	+	+

Because lesser omental hernia can cause acute intestinal obstruction and other severe clinical conditions, accurate diagnosis and timely treatment are essential to improve the clinical outcomes of patients. This is exceptionally important for elderly patients, such as the patient in the present case, who was 73 years of age. It is generally accepted that an intra-abdominal hernia impairs the relocation of the abnormal contents to the original location or the ability to self-repair. The intra-abdominal hernia can cause strangulated intestinal obstruction, intestinal necrosis, infectious shock, and even multiple organ failure, with a reported mortality rate as high as 75% [14]. Therefore, early diagnosis of intra-abdominal hernia and the performance of the appropriate surgical procedures (e.g., removal of ischemic intestinal fragments and repair of the orifice of intra-abdominal hernia) are particularly important. The characteristic symptoms of this condition include severe and acute abdominal pain. In the diagnosis of intra-abdominal hernia, the plain abdominal film has little value, while abdominal CT scanning is of value [15]. In fact, enhanced CT is able to determine the blood flow of the intestine and the shape of the mesenteric vessels, thereby improving the diagnosis accuracy of intra-abdominal hernia and assisting in an evaluation of the intestinal necrosis. Based upon the abdominal CT findings of the cases that were diagnosed preoperatively, including ours (Table 1), the specific features of abdominal CT in cases of lesser omental hernia are summarized as follows: (1) Dilated bowel loops are located in the ventral part of the stomach; (2) Mesenterium are gathered in the lesser curvature of the stomach, where the hernia ring is also present; (3) The stomach is forced to become distorted and relocated, and similar changes may occur in the surrounding organs (e.g., spleen, transverse colon). Besides, it is also important to explore the epidemiology of lesser omental hernia after total colectomy in particular the incidence in order to know if total colectomy increases the risk of occurrence of this type of hernia by chance or iatrogenic forms.

Taken together, the cause of lesser omental hernia can be congenital or acquired. As the use of abdominal surgery is continuously increasing, the risk of developing abdominal surgery-associated complications, such as lesser omental hernia, is on the rise. Thus, careful examination of the small omentum before the closure of the abdomen is expected to reduce the occurrence of these abdominal surgery-associated complications. The characteristic findings of abdominal CT in the present case and those of previous cases may help other clinicians to obtain accurate diagnoses of lesser omentum hernia in the future.

## Data Availability

All data and materials are available in case of a request.

## Abbreviations

CIC: Chronic idiopathic constipation (CIC)
CT: Computed tomography
IPAA: Ileal pouch-anal anastomosis
LA: Lactic acid
WBC: White blood cell
